# COVID-19 Ramifications: A Scientific Approach to Bridge the Existing Gap between COVID Vaccines Hesitancy and Effectiveness

**DOI:** 10.1055/s-0042-1760338

**Published:** 2023-01-13

**Authors:** Hassan Nagy, Maha Hameed, Faryal Khan, Edzel Lorraine Co, Unaiza Rauf

**Affiliations:** 1 Division of Research & Academic Affairs, Larkin Health System, South Miami, Florida, United States

**Keywords:** Covid-19, vaccine effectiveness, vaccine hesitancy, coronavirus

## Abstract

The 2019 coronavirus disease (COVID-19) has been a major dilemma all over the world since December 2019. Several types of COVID-19 vaccines were developed for public utilization to halt the widespread of the disease; however, vaccine hesitancy is one major factor that prevents a successful control of this pandemic. This study aimed to summarize the different kinds of available COVID-19 vaccines and their effectiveness, and to assess the associated factors regarding vaccine hesitancy of the general population to bridge the gap existing between the two factors. 1 3

## Introduction

Coronavirus was first detected in China in December 2020. The World Health Organization declared a coronavirus pandemic in March 2020. Despite vaccination and social distancing, coronavirus disease 2019 (COVID-19) cases are expected to grow. Although a lot of research and studies are done on COVID, there is still a lot to be discovered yet. 1 2 This review summarizes the epidemiology, history, clinical signs, and COVID vaccines' effectiveness and hesitancy among the population. We searched PubMed data looking for relevant articles with meta-analysis and systematic review. Coronavirus was discovered as the main cause of a cluster of pneumonia cases in Wuhan, a city in China's Hubei Province. Coronaviruses are enveloped positive-stranded ribonucleic acid (RNA) viruses. The closest RNA sequence similarity is to two bat coronaviruses. Bats are likely the primary source. All different types of variants, including Beta, Alpha, Delta, and Gamma, are involved in causing the corona pandemic. 3 The majority of the patients developed upper respiratory symptoms as a cluster of pneumonia. Fifty-seven percent of the patients reported gastrointestinal symptoms, and the researchers believe that this is because of the swallowed virus. Due to inconsistent reporting and lack of accurate data, a universal case–fatality rate of COVID-19 is yet to be established. 1

### Methodology

We conducted a thorough search of articles from common databases (PubMed, Scopus, EBSCOhost, ScienceDirect, and Google Scholar) with the following search terms: “Coronavirus 19,” “Covid-19,” “pandemic,” “vaccine effectiveness,” and “vaccine hesitancy.” Book articles, commentaries, and editorials were not included. The initial search yielded a total of 34 articles, with articles used for the final analysis after a thorough evaluation has been done.

Information from the gathered articles was utilized to assess the effectiveness of each available COVID-19 vaccine and the prevailing factors that are related to vaccine hesitancy.

## Epidemiology

**Fig. 1:**
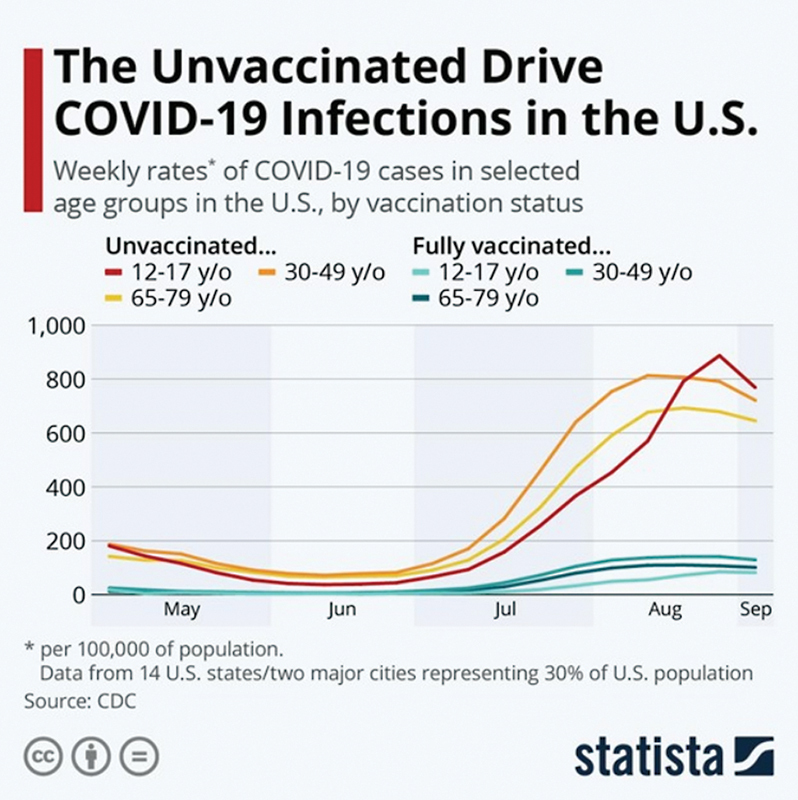
Weekly rates of coronavirus disease 2019 (COVID-19) in unvaccinated population in the U.S. (Source: Statista).

The COVID-19 pandemic has affected the world profoundly. As of February 2021, there were 395 million coronavirus cases and 5.74 million deaths. The documented numbers of coronavirus underestimate the overall burden of the pandemic as many cases go undiagnosed and underreported. COVID-19 can affect individuals of any age. The median age of hospitalized patients with confirmed COVID-19 ranges from 49 to 56 years. The middle-aged and elderly population seem to be the most commonly affected, and the virus affects males more often than females. The Centers for Disease Control and Prevention (CDC) reported 212.8 million people to be fully vaccinated and 89.8 million people received a booster dose ( Fig. 1 ). 1 3

**Fig. 2:**
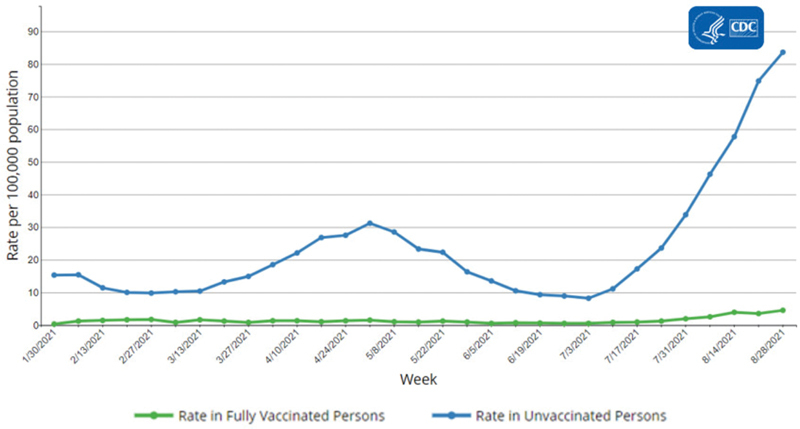
Hospitalization rates by vaccination status in adults (Source: Centers for Disease Control and Prevention [CDC]).

**Fig. 3:**
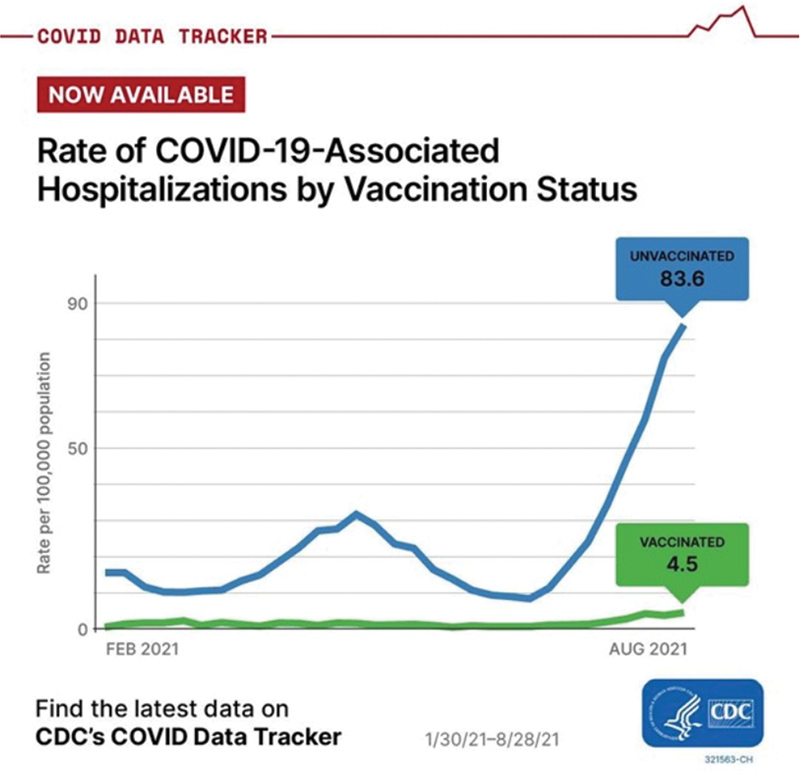
Rate of coronavirus disease 2019 (COVID-19)-associated hospitalizations by vaccination status (Source: Centers for Disease Control and Prevention [CDC]).

**Fig. 4:**
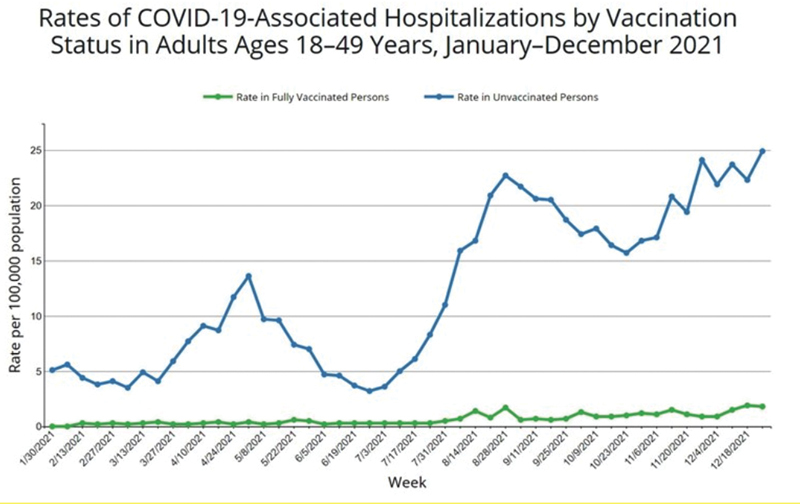
Rates of coronavirus disease 2019 (COVID-19)-associated hospitalizations by vaccination status in adults ages 18–49 years, January–December 2021 (Source: Centers for Disease Control and Prevention [CDC]).

Age-specific rates of COVID-19-associated hospitalizations are 14 times higher among unvaccinated adults aged 18 to 49 years, 15 times higher among unvaccinated adults aged 50 to 64 years, and 9 times higher among unvaccinated adults aged 65 years and older. Figs. 2 , 3 , 4 demonstrate current data on COVID-19-associated hospital admissions among vaccinated versus nonvaccinated people. 4

The virus mainly spreads via infected secretions and large aerosol droplets. Immunity develops soon after infection but wanes gradually over time. Reinfection is common, mainly because of waning immunity and also because of antigenic variation within species. Severe illness can develop in otherwise healthy individuals of any age, but it predominantly occurs in adults with advanced age and specific underlying medical comorbidities. The United States also has the most confirmed deaths globally, followed by Brazil, India, and Mexico. 2 3 5 6

## History

The incubation period for COVID is generally within 14 days following exposure, with most cases occurring 4 to 5 days after exposure. The range of associated symptoms reported to the CDC are: fever, myalgia, headache, dyspnea, sore throat, diarrhea, nausea/vomiting, loss of smell or taste, abdominal pain, rhinorrhea, conjunctivitis, delirium and general health decline, brain fog, cognitive impairment, pain, rash, mood change, and menstrual cycle irregularities. 3 5 6 7

## Physical Examination

Patients under investigation for COVID-19 should be evaluated in a private room with the door closed and asked to wear a surgical mask. Observe all standard contact and airborne precautions. The most common severe manifestation of COVID-19 upon the initial presentation is pneumonia. 7 The appearance of the patient infected with the severe acute respiratory syndrome (SARS) virus depends on the severity of the illness; the majority of the patients with mild disease may appear healthy. Patients will appear sick, lethargic, cyanotic with hypoxemia, and shortness of breath in severe disease and may also appear dehydrated with tachypnea (> 30 breaths/min) and hypotension. 7 8 Oxygen saturation, respiratory rate, chest X-ray, and, importantly, dyspnea help determine the need for invasive mechanical ventilation. They have increased vocal fremitus, diminished chest expansion at the affected side, dull percussion, and decreased breath sounds, rhonchi, and crackles. Nasal congestion and rhinorrhea are a relatively common finding. Skin examination may show erythema multiforme, maculopapular rash, urticaria, chickenpox, and livedo reticularis. Head and eye examination may show periorbital swelling due to acute kidney injury. The patient can present with COVID-associated myocarditis and heart failure with an S3 and regurgitant atrioventricular valves with murmurs. COVID-19-associated Guillain–Barre syndrome (GBS) and COVID-19-associated polyneuritis cranialis have also been reported. 7

The diagnostic approach to COVID-19 consists primarily of having a high index of clinical suspicion in order to conduct the testing. Symptomatic patients with suspected infection, as well asymptomatic patients who have a history of close contact with a COVID-19 positive individual, are being screened due to the need for early identification of the infection in living facilities that are at risk for severe disease (e.g., long-term care facilities), or need to be tested prior to surgical procedures or receiving immunosuppressive therapy. 9

In certain asymptomatic cases, however, the CDC suggests against retesting asymptomatic individuals that are either fully vaccinated and need to fulfill posttravel testing requirements or were previously diagnosed with COVID-19 within the past 3 months (due to the lower likelihood that a repeat positive test would truly represent active reinfection).

Nucleic acid amplification testing (NAAT), most commonly with a subsequent reverse-transcription polymerase chain reaction assay is the preferred initial diagnostic test for COVID-19, for the detection of SARS coronavirus 2 (SARS-CoV-2) RNA from the upper respiratory tract. 10 In situations where NAAT access is limited, too costly to use, or there is not enough time to wait for the result, antigen testing would be the second-best initial test. However, the sensitivity of antigen testing is lower than that of NAAT, thereby indicating the need for a negative antigen test to be confirmed with further additional testing.

The CDC recommends the following methods of upper respiratory tract specimen collection for the diagnosis of COVID-19: nasopharyngeal swab, a nasal swab from both anterior nares, nasal mid-turbinate swab, nasal or nasopharyngeal wash/aspirate, oropharyngeal swab, or saliva specimen (1–5 mL). 11

A positive NAAT for SARS-CoV-2 confirms the diagnosis of COVID-19, with no additional testing being necessary. Similarly, a single negative NAAT result is enough to exclude the diagnosis of COVID-19. In cases where the initial testing is negative, but the suspicion for COVID-19 remains (e.g., suggestive symptoms that cannot be explained otherwise), it could indicate a false-negative test that needs to be repeated.

In patients that present 3 to 4 weeks into the course of COVID-19 infection and have negative NAAT, checking a serologic test such as immunoglobulin G (IgG) test may be helpful. A reactive IgG can be suggestive of COVID-19, whereas a negative test would be indicative of the decreased likelihood of the infection.

As of February 2022, 61.4% of the entire world's population has received one dose of the COVID-19 vaccination with a total of 10.22 billion doses having been administered globally at the time of this review. _1_ Currently, the United Arab Emirates has achieved the highest vaccination rate, 94% of the country's population being fully vaccinated, and 99% having received at least one dose of the COVID-19 vaccine.

On the other side of the spectrum, there is a growing level of concern and hesitancy among the general population to get vaccinated. Several reasons have been identified in the literature including refusal to receive any vaccine in general, safety concerns, doubts regarding the efficacy, or need for the vaccine as a whole. 10

Several studies have emerged in demonstrating both the safety and efficacy of various COVID-19 vaccines, such as an efficiency of 95% noted in COVID-19 prevention with the Pfizer vaccine and 94.1% with Moderna. 11 12 The Pfizer COVID-19 vaccine was also noted to have a 100% vaccine efficacy rate in South Africa against the predominant Beta strain. 13

Another common misconception that has also contributed toward vaccine hesitancy is the belief that the COVID-19 vaccines are not effective against new strains of the SARS-CoV-2 virus, such as the recent emergence of the Delta variant. However, a recent study conducted in England comparing the effectiveness of different COVID-19 vaccines against the Alpha and Delta variant has suggested otherwise. 14

An absolute difference of 11.9% higher effectiveness of the Pfizer COVID-19 infection was found for patients with the Alpha variant as compared to the Delta variant, and a similar 18.7% higher effectiveness of the AstraZeneca vaccine was seen in the Alpha variant. However, this efficacy percentage was noted to be much smaller after patients had received their corresponding second vaccine dose suggesting the need for the completion of vaccination doses in the successful management of new emerging strains as well.

A systematic review and meta-analysis comparing the effectiveness of COVID-19 vaccines against the Delta variant compared with the Alpha variant demonstrated a similar slightly higher effectiveness of 10 to 20% for less severe outcomes in Alpha patients. However, the effectiveness of COVID-19 vaccines in hospitalization prevention did not differ between the Alpha and Delta variant positive patients. 15

### Types of COVID-19 Vaccines

The urgency to respond to the COVID-19 pandemic, forced the scientific community internationally to answer in terms of various therapeutics and vaccines, targeting different structural proteins of SARS-CoV-2 virus. Coronaviruses have an outer envelope consisting of spike (S) protein, envelope (E) protein, matriculates (M) protein, and a nucleocapsid (N) encasing a single-stranded positive sense RNA genome. Binding of S protein to its specific receptor enables cellular entry. Vaccines with antibodies against this S protein (such as mRNA-1273, BNT162b2, Ad26.COV2.S, AZD1222, Ad5-nCoV, Sputnik V, NVX-Cov2373, SCB-2019, INO-4800, etc.) or receptor-binding domain in S (such as ZF2001, BNT162b1, ARcoVax, etc.) can nullify this effect.

### Pfizer-BioNTech Comirnaty COVID-19 Vaccine

Name: Pfizer-BioNTech Comirnaty COVID-19 Vaccine 16

Manufacturer: BioNTech Manufacturing GmbH

Type of Vaccine: mRNA

Approved for: Age 5 and older

Mode of Administration: Intramuscular (Usually the Upper Arm)

Number of Doses: 2

*Mechanism of action* : The mechanism of action of RNA vaccines is important to understand how the most common COVID-19 vaccines work. The RNA vaccines closely resemble the antigen that the body encounters during the natural infection. The Pfizer vaccine has a special mechanism in that it has a 1-methyl-pseudouridine attached to the RNA which allows the RNA to be not as easily detected by the innate immune response while also increasing messenger RNA (mRNA) translation. The mRNA expressed codes for the spike protein of the COVID virus and is an important target of antibodies that neutralize the virus. These antibodies are produced by the host once it encounters the antigen. The vaccine RNA is also contained in lipid nanoparticles (LNPs) which allows for more efficient delivery into the cells. RNA is also very safe as it does not integrate into the subject's genome and is metabolized and removed via the body's natural mechanisms. 16

*Effectiveness* : 91.3% effective at preventing symptoms when infected with COVID-19, 7 days and up to 6 months after the second dose. 13

*Side effects* : The most common side effects in decreasing incidence are injection site pain (84.1%), fatigue (62.9%), headache (55.1%), muscle pain (38.3%), chills (31.9%), joint pain (23.6%), fever (14.2%), injection site swelling (10.5%), injection site redness (9.5%), nausea (1.1%), malaise (0.5%), lymphadenopathy (0.3%), severe allergic reaction (rare), and temporary one-side facial drooping (rare). 17

### Moderna

Name: Moderna COVID-19 Vaccine 17

Manufacturer: ModernaTX, Inc.

Type of Vaccine: mRNA

Approved for: 18 years and older

Mode of Administration: Administered intramuscularly in upper arm

Number of Doses: 2 (28 days apart) in the same arm. 16

Storage: –25°C

*Mechanism of action* : Moderna and Pfizer are both mRNA vaccines and work by the same principle. Moderna is a LNP-encapsulated modified mRNA-based vaccine. These mRNA particles, once inside the vaccine recipient, are translated into spike (S) viral proteins, which is essential for viral attachment and entry into the host cells. This spike protein then gets displayed on the cell surface for our immune system to be detected that it does not belong here and generate antibodies against it, and attack the spike protein the next time it sees one. 17

*Efficacy* : 94.1% effective in prevention for symptomatic SARS-CoV-2.

*Adverse effects* : 0.1% (myopericarditis), delayed injection-site reaction (1.1). 18

### Jansen

Name: Janssen (Johnson & Johnson) 19

Manufacturer: Janssen Biotech Inc

Type of Vaccine: Modified Human Adenovirus

Approved for: Ages 18 years and older

Mode of Administration: Administered intramuscularly

Number of Doses: Single dose

Storage Mechanism of Action: 2°C and 8°C (36°F and 46°F) 20

*Mechanism of action* : It is replication-incompetent viral vector vaccine, which contains genetic code for viral spike (S) protein expressed on its surface after entering human cells. The viral vector used here is based on a naturally occurring, low-prevalence human adenovirus. 19

*Efficacy* : Moving from day 14 onwards after a single dose efficacy for moderate to severe/critical COVID-19 cases was found to be approximately 67% and approximately 77% for severe/critical cases.

Vaccine efficacy of approximately 85% for severe/critical COVID-19 cases occurring from day 28 onward after receiving a single dose. 19

*Adverse effects* : Thrombosis with thrombocytopenia (97% nonserious and 3% serious), cerebral venous sinus thrombosis, fatigue (59.1%), pain (57.9%), headache (52%), fever and chills (34%), joint pain (26%), nausea (18%), diarrhea (9.4%), rash (1.9%), and GBS. 19

Name: Sputnik V (Gam-COVID-Vac) 21

Manufacturer: Gamaleya Research Institute

Type of Vaccine: Modified Human Adenovirus

Approved for: Ages 18 years and above

Mode of Administration: Ad26 is used in the first dose and Ad5 is used in the second dose given 3 weeks apart.

Storage: –18.5°C (liquid form), 2–8°C (dry form)

*Mechanism of action* : Heterogeneous adenovirus 26 (Ad26) and adenovirus 5 (Ad5) used as vectors for expressing the SARS spike (S) protein. This virus after entering cell express spike (S) protein but then stops, as it cannot initiate a reproductive infection. To respond this host immune system generates neutralizing antibodies that enables virus from binding to its receptors. 21 22

*Efficacy* : 91.6%.

*Adverse effects* : Pain in the injection site (56.9%), fatigue (50.9%), body pain (43.9%), headache (35.7%), fever (32.9%), joint pain (30.3%), chilling (29.8%), and drowsiness (20.3%). 21 22

### AstraZeneca Vaccine

Name: AstraZeneca Oxford 13 16

Manufacturer: AstraZeneca

Type of Vaccine: Modified Chimpanzee Adenovirus

Approved for: Age 18 and older

Mode of Administration: Intramuscular

Number of Doses: 2

*Mechanism of action* : The AstraZeneca vaccine employs a chimpanzee deoxyribonucleic acid (DNA) adenovirus that has been redesigned and never been uncovered to human populations and only generates an immune response to the viral protein encoded in the host DNA, rather than the adenovirus itself. 13 16

#### Effectiveness

*Side effects* : The most common side effects in decreasing incidence are injection site pain (84.1%), fatigue (62.9%), headache (55.1%), muscle pain (38.3%), chills (31.9%), joint pain (23.6%), fever (14.2%), injection site swelling (10.5%), injection site redness (9.5%), nausea (1.1%), malaise (0.5%), lymphadenopathy (0.3%), severe allergic reaction (rare), and temporary one-side facial drooping (rare). 16

## Differential Diagnosis 4 10 23 24 25

1. Community-acquired pneumonia
2. *Pneumocystis jirovecii* pneumonia
3. Interstitial pneumonia
4. Common colds
5. Influenza
6. Other coronavirus infections (SARS, Middle East respiratory syndrome)
7. Gastroenteritis
8. Bronchitis
9. Myositis
10. Arthritis
11. Urinary tract infection
12. Cervical lymphadenitis
13. Alcoholic hepatitis
14. Kawasaki disease
15. Viral meningitis
16. Bacterial meningitis
17. Autoimmune encephalitis
18. Viral encephalitis
19. Bacterial sepsis
20. Toxic shock syndrome
21. Appendicitis
22. Systemic lupus erythematosus
23. Vasculitis
24. Dengue fever
25. Venous thromboembolism
26. Oral herpes simplex virus-1
27. Erythema multiforme
28. Acute myocardial injury

## Discussion of Vaccines Effectiveness

With the increasing cases of COVID-19 worldwide, the discovery of vaccines is the most common goal of all drug companies to combat COVID-19. Several kinds of vaccines have been developed and tested for their safety, effectiveness, and efficacy. These available vaccines in the market have varying profiles, with evidence showing that all of them have acceptable short-term effects. Having a comprehensive background of each of these vaccines is very important to keep in mind to be able to decide which COVID-19 vaccine is best suited to be given to a patient considering also his previous medical history. In this way, we can maximize the effectiveness of a particular vaccine and minimize any untoward adverse effects that may arise, hence achieving our goal of herd immunity against COVID-19.

### Efficacy

All COVID-19 vaccines were found to have good efficacy and can substantially decrease the risk of contracting the disease, with the RNA-based vaccines possessing the highest efficacy of 94.29% while inactivated vaccines with the lowest one, but still, with more than 70% effectiveness. Blacks or African American races can experience a greater effect of the vaccine. Protein subunit vaccines have a recorded efficacy of 89%, viral vector vaccines with 79% efficacy, and inactivated vaccines with a relatively lower efficacy rate. 23 26

### Immunogenicity

Each COVID-19 vaccine has a different immunogenicity profile. BNT162b2 (Pfizer-BioNTech) has shown in a phase I/II randomized clinical trial to have comparable antibody production to patients who had been infected with moderate SARS-CoV-2. It is more immunogenic in adolescents and children as compared to young adults. Following vaccination with BNT162b2, neutralizing antibody titers decline with time, roughly over 6 months. In a phase I clinical trial, mRNA-1273 (Moderna) also demonstrates antibody responses comparable with healthy individuals and can decline over 6 months. As compared with BNT162b2, the antibody titers produced by mRNA-1273 are higher. Just like with BNT162b2, neutralizing antibody titers are retained against all variants of concern, although this kind of vaccine generates a lower response against Beta and Delta variants. With Ad26.COV2.S (Janssen/Johnson & Johnson), the neutralizing antibody responses were found to be stable over 8 months following a single- or double-dose regimen. Retained neutralizing antibodies against Beta and Delta variants are slightly lower. ChAdOx1 nCoV-19/AZD12222 (University of Oxford, AstraZeneca, and Serum Institute of India) can produce higher antibodies following two and three doses of the vaccine. However, there is a possibility of immunity evasion against Beta and Delta variants. Other vaccines, such as Ad5-based COVID-19 vaccine (CanSino Biologics), Gam-COVID-Vac/Sputnik V (Gamaleya Institute), WIV04 and HB02 (Sinopharm), CoronaVac (Sinovac), Covaxin (Bharat Biotech/Indian Council of Medical Research), ZyCov-D (Zydus Cadila), and NVX-CoV2373 (Novavax), have shown to have neutralizing antibodies lower than other COVID-19 vaccines following vaccination. 23

### Safety and Adverse Drug Reactions

Another consideration for choosing a COVID-19 vaccine is safety. In a meta-analysis study of clinical trials of CDC, there is a vast spectrum of adverse drug reactions (ADRs) which involve multiple systems. The most commonly observed ADRs are headache, fatigue, and pain. The severity of these ADRs is just in the tolerable range from grades 1 to 2, with only fever reaching up to grade 4. Majority of these symptoms self-resolve after vaccination, even without any intake of medication. RNA-based vaccines have recorded ADRs reaching over 80% while the protein subunit vaccines have only 57% of which a grade 3 ADR noted to be the highest incidence of these ADRs. Viral vector vaccines have been reported to have thromboembolic events, myocarditis, or pericarditis. There were no reported ADRs associated with inactivated vaccines yet. The timing of onset of most ADRs developed within 1 week after vaccination. 22

The use of nonsteroidal anti-inflammatory drugs (NSAIDs) after getting vaccinated with any of the available COVID-19 vaccines has no significant effect on the efficacy of vaccines. Patients who use NSAIDs did not have an increased risk of contracting severe COVID-19 disease or death which is comparable to those who were not using NSAIDs at all. 22

#### Deterrence and Patient Education

Vaccination against COVID-19 is very important in order to get protection from acquiring severe diseases, not only for one's own sake but also for the health of every member of the family. Everyone is eligible to receive the COVID-19 vaccines including those who are pregnant, immunocompromised, and children unless their health care provider has advised them otherwise. Booster doses are needed in most people to enhance the effect of the vaccine, as the protection against COVID-19 can wane over time. 24 27 Side effects of the vaccine can be observed for a day or two. These may include headache, soreness of injection site, fatigue, body malaise, fever, or chills. If symptoms persist for more than 3 days, it is advised to contact the health care provider. The use of proper wearing of masks is strongly advised as well as frequent hand washing techniques. Observance of social distancing is also important to minimize the acquisition of the said virus. 25

#### Enhancing Health Care Team Outcomes

This pandemic has brought a big uncertainty into the lives of people which pushed the majority to doubt and rethink the future. Physical and mental states are being put at stake while scientists are still on the way to discovering the solution to end the pandemic. The surge of COVID-19 cases is alarming and therefore, the importance of great interdisciplinary teamwork at every level is so relevant in terms of good leadership, continuous communication, transparent decision-making, flexibility, and support to one another. The administrators and staff of the hospital have a big role in ensuring the availability of medical supplies and harmonious transfer of other facilities to render the best care possible to patients. Physicians in every field of specialty and nurses are very important in the comprehensive care of every patient. Nutritionists are needed to cater to the food requirements of every patient. The phlebotomists, medical and radiological technicians, pharmacists, and dentists are equally important to maintain the good health of patients. With an effective collaboration among all these health care providers, the complexity of this challenging crisis can be addressed successfully. 28 29

#### Limitations and Recommendations

This study is a scoping review limited to search for COVID-19 vaccines and possible causes of the hesitancy of their use. It is highly recommended to provide public dissemination of information regarding COVID-19 vaccine literacy including all health care workers to effectively combat COVID-19.

## Conclusion

The overall health burden brought about by COVID-19 has been severely detrimental to many lives of people. Several types of COVID-19 vaccines have been developed worldwide to halt the rapid spread of the disease and provide immunity to everyone as soon as possible. 30 31 However, vaccine hesitancy by some individuals is still an important issue to address as this hinders the embracement of massive vaccination. A holistic approach must be done to gain the public's trust in vaccination and achieve herd immunity. This circumvents all social, cultural, and political views of each country and the current status of their health care system. Targeting the underlying reasons for vaccine hesitancy at individual and social levels and correcting any misconceptions that one might have about the use of COVID-19 vaccines can then bridge the gap between vaccine effectiveness and hesitancy. 32 33
